# Influenza vaccination in Austria from 1982 to 2023—Decades of consistent ignorance and refusal

**DOI:** 10.3389/fpubh.2025.1692437

**Published:** 2025-12-04

**Authors:** Ursula Kunze, Richard Felsinger, Ernest Groman

**Affiliations:** Department of Social and Preventive Medicine, Center for Public Health, Medical University of Vienna, Vienna, Austria

**Keywords:** influenza, vaccine-preventable disease, vaccination, vaccination use, vaccination rate, Austria

## Abstract

**Objective:**

Influenza still tops the list of vaccine-preventable diseases and deaths in Austria. For more than four decades, Austria has grappled with persistently low rates of influenza vaccination, despite the clear availability of vaccines and recommendations from health authorities. This phenomenon is characterized by widespread public hesitation and systemic challenges within the healthcare system, resulting in a pronounced gap between vaccination campaigns and actual uptake. An average epidemic results in approximately 350,000–400,000 cases and 1,300 deaths annually in Austria. Despite this, vaccination rates remain alarmingly low, with coverage slightly above 10%, making it one of the lowest worldwide. Three earlier publications have reported the Austrian influenza vaccine use over several time periods between 1982 and 2015. This study extends these findings, analyzing data from eight additional influenza seasons (2016/17 to 2023/24), offering insights into over four decades of influenza vaccine utilization in Austria.

**Material and methods:**

Vaccine use data, presented as vaccine dose distributions per 1,000 population and vaccination rate percentages, from international studies and from the Austrian market only, were used.

**Results:**

Austria has consistently ranked among the countries with the lowest vaccine distribution rates; the number peaked in 2006 at 142 doses per 1,000. Since 2007, there has been a steady decline to 62 doses per 1,000 in the 2015/16 season, like levels observed in the mid-1990s. In the following years, a slight increase was observed to 85 doses per 1,000 in the 2019/20 season. During the 1st year of the COVID-19 pandemic, the number of doses distributed more than doubled to 221 doses per 1,000, before falling again in subsequent seasons.

**Conclusion:**

Despite comprehensive vaccination recommendations, influenza vaccination remains significantly undervalued by the Austrian population and many sectors of the health care system.

## 1 Introduction

According to the World Health Organization, seasonal influenza leads to approximately one billion infections worldwide each year, with 3–5 million cases classified as severe and 290,000–650,000 resulting in respiratory-related deaths (1). Similarly, a recent systematic literature review estimated 3.2 million cases of severe illness (hospitalizations) per annum worldwide (2). Within the European Union/European Economic Area (EU/EEA), around 50 million symptomatic influenza cases occur annually, causing 15,000–70,000 deaths (3). In Austria, seasonal influenza results in an estimated 350,000–400,000 infections and about 1,300 deaths annually (ranging between 360 and 4,000) (4). Hence, influenza-related deaths rank among the leading vaccine-preventable causes of death in the country.

In European comparison the Austrian influenza vaccination recommendations are quite extensive (5). The general recommendation for everyone from 6 months of age has been already established in 2002 (in the US not until 2010). Bulgaria and Estonia are the only European countries recommending the vaccination for adults over 18 years (6).

Despite the broad vaccination recommendations, the influenza vaccination rate (VR) in Austria has consistently been very low, averaging just 10%. This places Austria among the countries with the lowest vaccination rates worldwide. There are hardly any Austrian group- or age-specific vaccination coverage data available, only a few observational studies reveal some evidence (7–10). According to the latest wave of the cross-sectional Austrian Health Interview Survey, conducted in 2019, more than two-thirds (69.8%) of respondents aged 15 years and older said they had never had a flu vaccination and among the vulnerable older population (aged 60 and over), only 16.2% reported having had a flu vaccination in the year before the survey (11).

Three earlier publications have reported the Austrian influenza vaccine use over several time periods between 1982 and 2015 (12–14). The present paper reveals data of eight additional influenza seasons from 2016/17 to 2023/24, hence, covering a period of more than 40 years of influenza vaccine use (1982 to 2023).

## 2 Material and methods

The estimation of vaccination coverage through dose distribution analysis has been conducted by various research groups since the early 1990s. Four studies conducted by independent researchers and the European Scientific Working Group on Influenza, spanning from 1980 to 2003 and calculating the annual distribution of influenza vaccine doses (excluding those returned to the manufacturer), have laid the groundwork for understanding the global epidemiology of influenza vaccination (15–19).

Subsequently, the International Federation of Pharmaceutical Manufacturers and Associations Influenza Vaccine Supply (IVS) Task Force, whose members altogether produce and deliver most of the world's seasonal influenza vaccines, established an analysis procedure to provide ongoing regional and global data (19). This work, covering a study period up to 2015, finally illustrated data on the seasonal influenza vaccine distribution in more than 200 countries (20, 21). In all these studies, vaccine use has consistently been expressed as the number of doses distributed per 1.000 population, while population data were obtained from the United Nations' statistics database (21). Austrian data from these studies (15–20), covering the period from 1982 to 2015, has been summarized in previous papers (12–14).

This update extends the analysis to include the years 2016 to 2023, using data exclusively provided by the Austrian Association of Vaccine Manufacturers (22). The total of distributed doses per influenza season—minus returns—was collected and reported to an independent law office by the pharmaceutical companies. The VR percentage was calculated by using Austrian population data (23) and the total of distributed doses {%vaccinated population = [(distributed doses – returns)/Austria population] × 100}. As the actual number of used doses is unknown, the results reflect the theoretical maximum VR in the population.

## 3 Results

**Figure 1:**
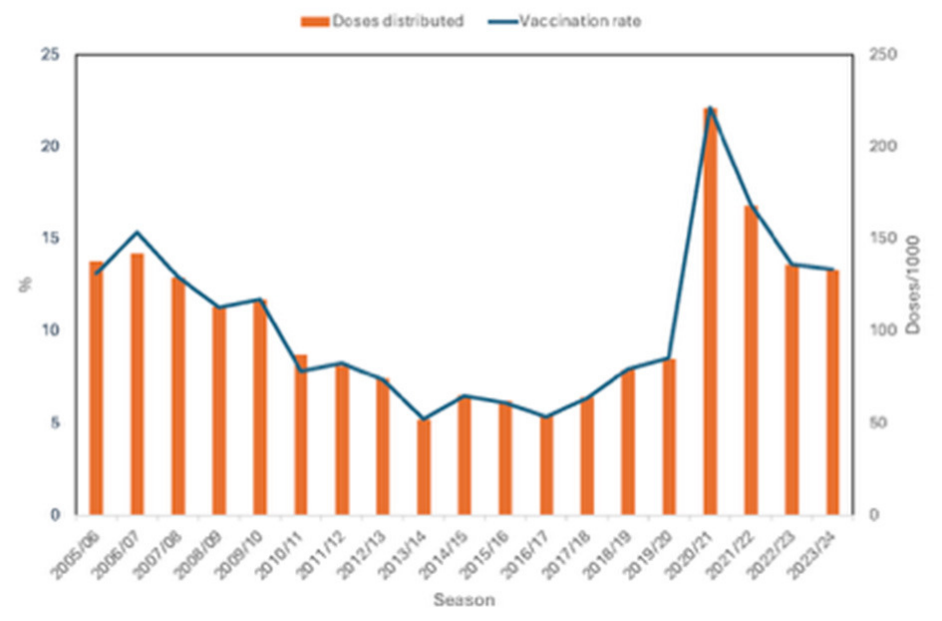
Annual number of doses of influenza vaccine distributed [n/1.000 population (15–21)] and estimated theoretical maximal vaccination rate in %, Austria, 2005/06-2023/24 (22); Population data: Statistics Austria (23). There are discrepancies in some years (esp. 2006 and 2010), which can be explained by the different research methods.

The Austrian data of four study periods (1980–1992, 1993–1995, 1996–2003, and 2004–2011) have been described extensively in three earlier papers (12–14). In summary, influenza vaccine use increased significantly between 1982 and 2003, starting from a very low level of 20 doses per 1,000 population and rising to 127 doses per 1,000. The peak distribution was recorded in 2006/07, with 142 doses per 1,000, from 2007/08 onward, vaccine use steadily declined to 81 doses per 1,000 by 2011, followed by a further drop to just 62 doses per 1,000 during the 2015/16 season (Figure 1)—returning to levels comparable to the mid-1990s (13, 14).

Over the next several years a slight increase was observed up to 85 doses per 1,000 in the 2019/20 season. The season 2020/21 was a remarkable one as the number of distributed doses more than doubled to 221 doses per 1,000. This was the first year of the COVID-19 pandemic and the first time when extensive social marketing activities for influenza vaccination have been undertaken. This “COVID-19” effect was of very short duration as the number of doses fell again in the following seasons (to 168 doses per 1,000 in the 2021/22 season, 136 doses per 1,000 in the 2022/23 season and 133 doses per 1,000 in the 2023/24 season). Figure 1 illustrates the numbers of doses as well as the estimated VR of the Austrian population for the seasons 2005/06 to 2023/24. Hence, the current VR of 13% remains among the lowest worldwide.

## 4 Discussion

There is a wide international consensus on the need to increase influenza vaccination rates (24). High seasonal influenza vaccination coverage is important because of several reasons, for example the protection of vulnerable groups, establishment of herd immunity or for pandemic preparedness with respect to increase the production capacity for influenza vaccines. Although capacity of and accessibility to influenza vaccines have been improved over the last years, the world remains unprepared to adequately respond to an influenza pandemic (25).

Between 2004 and 2019, the total number of doses administered worldwide increased by 103%, from around 262 million to 531 million, with variations across WHO regions (and largely driven by increase in the Americas). In the EURO region, the share of doses distributed has declined from 34% in 2004 to 27% in 2019 (25). In the EU, the number of doses distributed in 2019 was 153 doses per 1,000, which was the highest since the peak of 167 doses per 1,000 in 2009, and still 41% lower than the rate in the Americas. The most notable increase occurred in the EU region between 2017 and 2018 when distributes doses rose by 49% and between 2018 and 2019 with a further 11% increase (25).

In Austria, following a rise in influenza vaccine usage from a very low starting point between 1982 and 2006, a consistent decline was observed, reaching the levels seen in the mid-1990s (62 doses per 1,000 in the 2015/16 season) (12, 13). During the next few years, the numbers showed a slight increase up to 85 doses per 1,000 in the 2019/20 season. The peak was reached in the season 2020/21 when the number of distributed doses more than doubled to 221 doses per 1,000. Prior to the COVID-19 pandemic, the impact of influenza on population health, as well as its prevention, has received little to no attention in health policy. Hence, in the winter of 2020/21, there was great fear of a possible collapse of the health care system if COVID-19- and influenza patients needed intensive care at the same time. Therefore, for the first time ever, extensive social marketing activities for influenza vaccination, conducted and fully financed by the government, have been undertaken. The access to the vaccines was very easy, specially constructed vaccination centers were available (for example in shopping centers, which has never been done before) and the concomitant administration of influenza vaccines with COVID-19 vaccine doses was provided. After this first “Covid winter” the numbers of distributed doses dropped again to 168 doses per 1,000 (2021/22), 136 doses per 1,000 (2022/23) and 133 doses per 1,000 (2023/24).

When we demonstrate the development of VR, the data show a large proportion of the Austrian population has not been vaccinated, and there has been no significant change since 2005. In 2005, the VR was 13%, after a slight rise to 15% it decreased over the following years and in 2013 the lowest VR ever was reached with 5%. Until 2019 the VR ranged between 6% and 8.5%, when in 2020, the “Covid season,” it jumped to 22%. Then the VR fell again to about 13%.

There are only few Austrian data available regarding VR in specific risk groups like the older population or health care workers—all revealing low VR in the range of 10 to 42%—and no data regarding children and pregnant women (7–10). A recent review demonstrated a VR of 42.5% in health care workers in Europe (26).

According to the ECDC, after nearly a decade of stagnating or declining VR, Europe revealed an increase in uptake levels, which was observed for older age groups, persons with chronic conditions and healthcare workers (6). In the nine countries reporting vaccination rates for the 2020/21 season, the median VR in adults aged 18 years and above was 20% (6.4% Czechia, 8.1% Lithuania, 11.7% Slovenia, 18% Sweden, 23% Denmark, 26.4% Italy, and 27% Norway), which was 5% higher than during the 2018/19 season (5.4% Slovenia, 5.5% Czechia, 15% Sweden and Denmark, 16.4% Italy, and 20% Norway) (6). Compared to previous years, VR raised in all countries during the 2020/21 season. Norway recorded the greatest increase over the three seasons, from 20% in 2018/19 to 27% in 2020/21. In Sweden, Slovenia, Lithuania, and Czechia, on the other hand, VR stayed below 20% throughout the three seasons (6).

Like in Austria, the rise in vaccination rates for the 2020/21 influenza season is likely traced to the context of the COVID-19 pandemic, especially during the early part of the season before the COVID-19 vaccine was present (6). Considerably efforts were undertaken to improve the approach to influenza vaccines and were like those in Austria (e.g., fully financed vaccination programs with no cost for citizens, awareness campaigns, and co-administration of both vaccines) (6, 27).

Although a limited number of countries reached or approached the EU target vaccination rates (75% coverage for older populations and individuals with chronic medical conditions), overall uptake remains suboptimal and well below public health targets (6, 28). In the population aged 65 and older of reporting 19 countries, the median VR was 59% during the 2020/21 season, with values ranging from 4.5% in Latvia to 75% in Denmark. This is an increase from 51% in the 2018/19 season, where the range was from 8.1% in Latvia to 68.5% in Ireland. In all countries, the VR in older adults rose during the 2020/21 season in contrast to the 2018/19 season, except in Latvia, where it declined, and Slovakia, where it remained unchanged. The only country to reach a 75% VR was Denmark, marking a sharp rise from 52% in the two preceding seasons (6).

Both the Austrian public and health policies, as well as some areas of the medical sector, have shown a notable disregard for the prevention and control of influenza in the past. The possible reasons (e.g. absence of officially published vaccination coverage target rates for risk groups, negative attitudes among some health-care workers, generally low vaccination rates in adults, and lack of vaccine recommendations from a trusted (family) doctor) behind this development have been described extensively in earlier papers (12–14). Many of those reasons are of the same topicality as they were in the past. Perception of the severity of the disease and the importance of vaccination remain limited, the impact of influenza is underestimated and neglected, especially by the Austrian population, despite all efforts that have been undertaken during the first COVID-19 winter 2020/21. Before the COVID-19 pandemic, health policy was hardly interested in the topic. Studies indicate that knowledge levels and awareness of influenza and vaccination additionally remain low among some healthcare workers, especially nursing staff (8).

Furthermore, the implementation of a first time countrywide public vaccination programs in the season 2023/24 did not lead to a measurable increase in vaccination rates. Recommendations and proposals for improvement should be addressed in a future paper.

## Data Availability

Publicly available datasets were analyzed in this study. This data can be found here: https://web.oevih.at/daten_und_fakten/marktforschung-influenza/, https://www.statistik.at/statistiken/bevoelkerung-und-soziales/bevoelkerung/bevoelkerungsstand/bevoelkerung-im-jahresdurchschnitt.
